# Development and characterization of phage display-derived anti-toxin antibodies neutralizing TcdA and TcdB of *Clostridioides difficile*

**DOI:** 10.1128/spectrum.05310-22

**Published:** 2023-09-05

**Authors:** Hamideh Raeisi, Masoumeh Azimirad, Hamid Asadzadeh Aghdaei, Amir-Hassan Zarnani, Jalal Abdolalizadeh, Abbas Yadegar, Mohammad Reza Zali

**Affiliations:** 1 Foodborne and Waterborne Diseases Research Center, Research Institute for Gastroenterology and Liver Diseases, Shahid Beheshti University of Medical Sciences, Tehran, Iran; 2 Basic and Molecular Epidemiology of Gastrointestinal Disorders Research Center, Research Institute for Gastroenterology and Liver Diseases, Shahid Beheshti University of Medical Sciences, Tehran, Iran; 3 Department of Immunology, School of Public Health, Tehran University of Medical Sciences, Tehran, Iran; 4 Immunology Research Center, Tabriz University of Medical Sciences, Tabriz, Iran; 5 Gastroenterology and Liver Diseases Research Center, Research Institute for Gastroenterology and Liver Diseases, Shahid Beheshti University of Medical Sciences, Tehran, Iran; Tainan Hospital, Ministry of Health and Welfare, Tainan, Taiwan

**Keywords:** *Clostridioides difficile*, TcdA, TcdB, single-chain fragment variable, neutralizing antibodies, scFv, molecular docking

## Abstract

TcdA and TcdB are known as the major virulence attributes of *Clostridioides difficile*. Hence, neutralizing the TcdA and TcdB activities can be considered as an efficient therapeutic approach against *C. difficile* infection (CDI). In this work, we utilized phage display technique to select single-chain fragment variable (scFv) fragments as recombinant antibodies displayed on the surface of phages, which specifically target native TcdA, or TcdB (nTcdA and nTcdB), and their recombinant C-terminal combined repetitive oligopeptide (CROP) domains (rTcdA and rTcdB). After three rounds of biopanning, abundance of phage clones displaying high reactivity with TcdA or TcdB was quantified through enzyme-linked immunosorbent assay (ELISA). Furthermore, selected scFvs were characterized by cell viability and neutralization assays. The gene expression of immunological markers, IL-8 and TNF-α, was examined in treated Caco-2 cells by RT-qPCR. The epitopes of neutralizing scFvs were also identified by molecular docking. Totally, 18 scFv antibodies (seven for TcdA and 11 for TcdB) were identified by ELISA. Among selected scFvs, two clones for TcdA (rA-C2, A-C9) and three clones for TcdB (rB-B4, B-F5, B-F11) exhibited the highest neutralizing activity in Caco-2 and Vero cells. Moreover, the cocktail of anti-TcdA and anti-TcdB antibodies notably decreased the mRNA expression of TNF-α and IL-8 in Caco-2 cells. Molecular docking revealed that the interaction between scFv and toxin was mostly restricted to CROP domain of TcdA or TcdB. Our results collectively provided more insights for the development of neutralizing scFvs against *C. difficile* toxins using phage display. Further research is needed to meticulously evaluate the potential of scFvs as an alternative treatment for CDI using animal models and clinical trials.

**IMPORTANCE:**

Targeting the major toxins of *Clostridioides difficile* by neutralizing antibodies is a novel therapeutic approach for CDI. Here, we report a panel of new anti-TcdA (rA-C2, A-C9) and anti-TcdB (rB-B4, B-F5, and B-F11) recombinant antibody fragments (scFvs) isolated from Tomlinson I and J libraries using phage display technique. These scFv antibodies were capable of neutralizing their respective toxin and showed promise as potential therapeutics against TcdA and TcdB of *C. difficile* in different *in vitro* models. In addition, *in silico* analysis showed that at least two neutralization mechanisms, including inhibiting cell surface binding of toxins and inhibiting toxin internalization can be proposed for the isolated scFvs in this work. These findings provide more insights for the applicability of specific scFvs toward *C. difficile* toxins at *in vitro* level. However, further research is required to evaluate the potential application of these scFvs as therapeutic agents for CDI treatment in clinical setting.

## INTRODUCTION

*Clostridioides difficile*, formerly named *Clostridium difficile*, is an anaerobic Gram-positive spore-forming bacillus. *C. difficile* infection (CDI) is known to be the major cause of nosocomial infections following antibiotic therapy worldwide (1). In recent decades, the incidence of CDI has been increasing in both adult and pediatric populations, and the surveillance data from 2017 estimated the number of CDI about ~223,900 cases, 12,800 deaths, and $1 billion healthcare cost in the United States (2). Two homologous exotoxins, toxin A (TcdA, molecular weight: 308 kDa) and toxin B (TcdB, molecular weight: 270 kDa), are the major virulence factors of *C. difficile*, which contain four functional domains, each of which plays a role in a multi-step mechanism of entry (3). The most important domain of each toxin is its C-terminus, which includes a domain with combined repetitive oligopeptide repeats (CROP), where long repeats (LRs) and short repeats (SRs) support the binding of the toxin to the cell membrane and its internalization by receptor-mediated endocytosis (3, 4). Each of TcdA and TcdB can independently cause a wide spectrum of clinical symptoms associated with CDI in humans (5). These toxins share similar pathogenic mechanisms affecting intestinal epithelial cells (IECs), inactivate Rho/Ras proteins, which lead to a loss of mucosal barrier integrity, intensive inflammation, diarrhea, pseudomembrane colitis (PMC), or even, in some cases, death (6, 7).

Recommended therapy for treating mild-to-moderate CDI includes antibiotic administration, in particular metronidazole, vancomycin, or fidaxomicin (8, 9), which consequently can lead to the disruption of the normal gut microbiota and development of antibiotic-resistant strains of *C. difficile*. Additionally, antibiotic therapy is an alarming factor for an increased number of CDI cases and a serious burden to public health in the last two decades (8, 10, 11). Presently, there is an urgent need for discovering novel and alternative treatment strategies for the effective management of CDI. Recently, antibodies with potent neutralizing activity have been introduced as promising therapeutic agents against CDI (12). According to the role of *C. difficile* toxins in disease development, most efforts for CDI treatment have focused on the production of therapeutics that can specifically prevent or modulate the pathologic effects of TcdA or/and TcdB on IECs (13 – 16). So far, two antibodies, actoxumab and bezlotoxumab, have been introduced for neutralizing the CROP domain of *C. difficile* toxins (17). Actoxumab is an anti-TcdA antibody that has not been approved for clinical use yet, whereas bezlotoxumab is an anti-TcdB antibody and gained FDA approval for treating recurrent CDI (rCDI) (17, 18). However, considerable amount of research is still ongoing in this area (19, 20). Among antibodies, recombinant antibody fragments, that is, fragment antigen binding (Fab), single-domain antibodies (sdAbs), and single-chain fragment variable (scFv) have received much attention in recent years (14, 16, 20, 21). The Fab contains constant and variable regions of the heavy (C_H1_ and V_H_) and light (C_L1_ and V_L_) chains, which are connected by interchain disulfide bridges (12). The sdAbs are very small in size and composed of V_H_ chain and devoid of V_L_ chain (20). The scFv is the most favorite type of recombinant antibodies that is constructed from V_H_ and V_L_ domains linked by a glycine-serine flexible linker. These fragments contain six specific zones, known as complementary-determining regions (namely CDR L1-L3 and CDR H1-H3), that interact with different antigenic determinants of an antigen, also known as epitopes (12, 22).

Phage display has been applied as the most common technique for generating scFv antibodies, and is considered as an alternative powerful platform for the selection of specific monoclonal antibodies (21). In principle, antibody production in phage display technology exploits phagemids containing a variety of genes encoding scFv fragments, which are fused with a coat protein (pIII or pVIII) of filamentous phages (M13) to be displayed on the surface of phage particles (21, 22). The selection of specific scFvs is carried out by *in vitro* screening of libraries through antigen-binding assays such as enzyme-linked immunosorbent assay (ELISA) or functional assays such as neutralization assay (12). This strategy has been proven to be a reliable, robust, cost-effective, rapid, and efficient approach for discovering and developing human therapeutic antibodies against various antigens, especially microbial toxins (12, 23, 24).

In this study, we describe the isolation of a panel of recombinant antibodies targeting native TcdA and TcdB, and their entire CROP domains from Tomlinson I and J naïve scFv libraries (MRC, Cambridge, England, ReIn_0017) using phage display technique. The sensitivity and specificity of screened antibodies to detect toxins were determined by ELISA. Additionally, the toxin-neutralizing activity and the modulatory effects of selected antibodies on the expression level of genes involved in inflammation, individually or as cocktail, were assessed under *in vitro* conditions.

## RESULTS

### Screening of phage libraries toward TcdA and TcdB by affinity selection

*C. difficile* strains TcdA-positive and TcdB-negative *C. difficile* RT084 (A^+^B^-^) and TcdA-negative and TcdB-positive *C. difficile* RT017 (A^-^B^+^) were used for obtaining native TcdA (nTcdA) and native TcdB (nTcdB), respectively. The purification of nTcdA and nTcdB was successfully confirmed by ELISA. Moreover, to achieve higher expression, the CROP domains of TcdA (1868–2704 aa) of *C. difficile* RT084 and TcdB (1802–2366 aa) of *C. difficile* RT017 were recombinantly expressed in *Escherichia coli* Rosetta strain (DE3) and purified using immobilized-metal affinity chromatography (IMAC). The results of the SDS-PAGE and western blotting confirmed the acceptability of the expression and purification of recombinant TcdA (rTcdA) and TcdB (rTcdB) (Fig. S1 at https://figshare.com/s/54a86cbfe656e260a75f). Based on results obtained by bicinchoninic acid (BCA) protein assay, the concentration of purified native and recombinant toxins was estimated about 390, 350, 700, and 850 µg/mL for nTcdA, nTcdB, rTcdA, and rTcdB, respectively.

**Fig 1:**
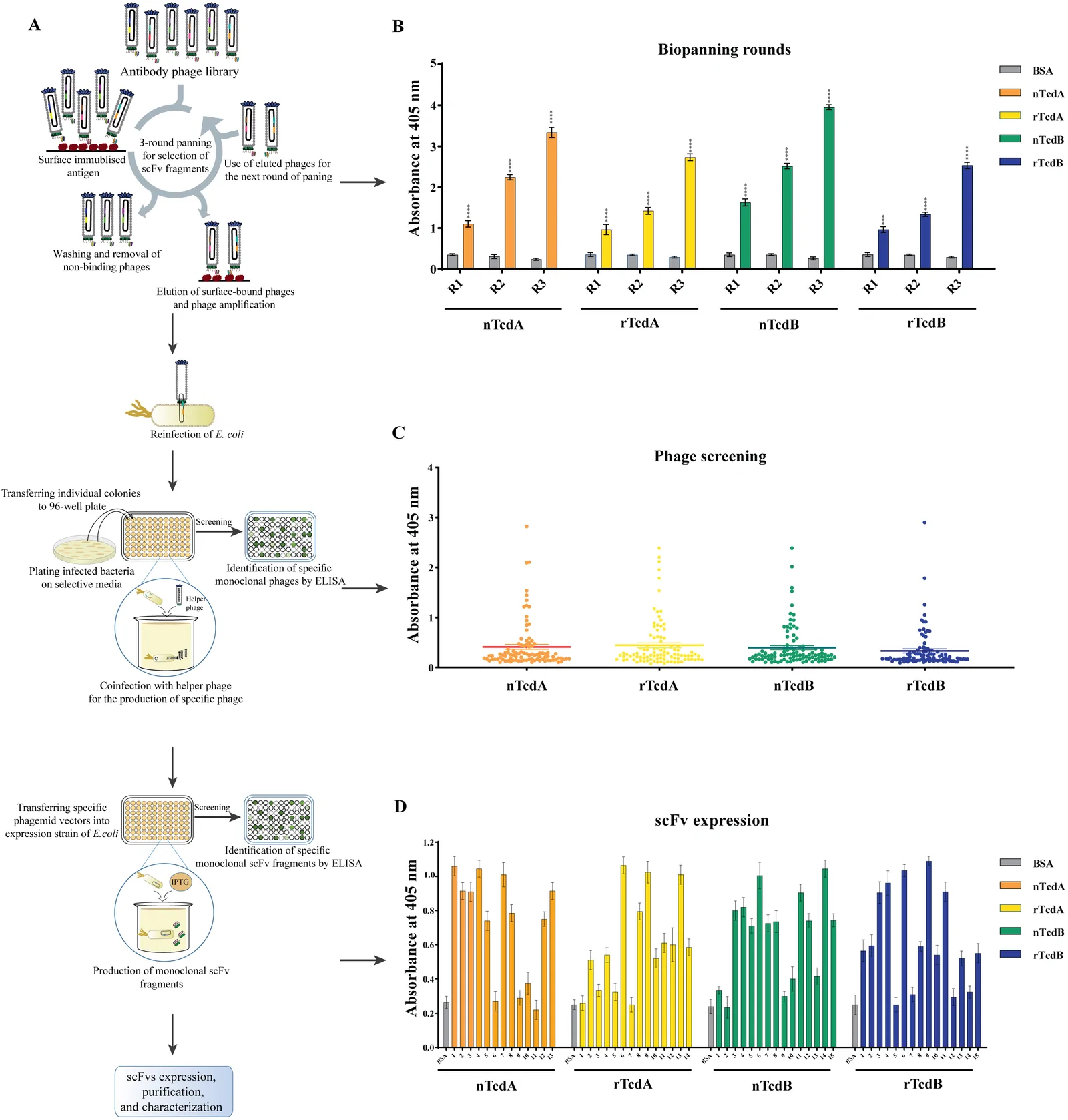
(**A**) Schematic overview of the experimental setup of the present study. (**B**) Enrichment of Tomlinson I and J scFv libraries against native TcdA (nTcdA) and TcdB (nTcdB) from *C. difficile* (RT084, RT017, respectively) as well as recombinant CROP domain fragment of TcdA (rTcdA) and TcdB (rTcdB) through three rounds of biopanning. (**C**) Phage-ELISA for detecting the binding activity of 96 randomly selected phage clones after three rounds of biopanning against their respective antigen. (**D**) scFv-ELISA for screening high-affinity clones to nTcdA or nTcdB. BSA was used as negative control. A *P* value of <0.05 was considered significant (****P < 0.0001) by unpaired Student’s *t* test and one-way ANOVA statistical analysis.

To select antibodies against nTcdA, nTcdB, rTcdA, and rTcdB, two naïve human scFv libraries I and J were used and three rounds of biopanning were performed on each of the four antigens. A schematic overview of the phage display method is presented in Fig. 1A. As indicated in Table 1, the number of recovered phages gradually increased after each round of biopanning. As expected, the determination of phage recovery rate indicated an increase of 644-fold, 725-fold, 611-fold, and 531-fold for the number of phages isolated in the last round of biopanning compared with the first round for nTcdA, nTcdB, rTcdA, and rTcdB, respectively. These findings were in agreement with the results of the polyclonal phage-ELISA performed after each round of biopanning (Fig. 1B), which showed a significant increase in the specificity of eluted phages toward different antigens during three rounds of biopanning (*P* < 0.001), whereas no reaction was found with bovine serum albumin (BSA) as negative control. These results reveal an obvious enrichment of phages with high affinity against antigens.

**TABLE 1:** Titers of Tomlinson I and J phage libraries eluted after biopanning processes^*a*^

Target	Round 1	Round 2	Round 3
nTcdA	4.5 × 10^4^	6.3 × 10^5^	2.9 × 10^7^
rTcdA	3.6 × 10^4^	4.8 × 10^5^	2.2 × 10^7^
nTcdB	5.1 × 10^4^	5.7 × 10^5^	3.7 × 10^7^
rTcdB	3.2 × 10^4^	3.4 × 10^5^	1.7 × 10^7^

^*a*^ Input phage per round: 10^13^ pfu/mL.

### Determination of the binding activity of monoclonal phage clones to TcdA or TcdB

The ability of monoclonal phages to detect toxins was examined by phage-ELISA, in which selected phage clones were used as primary antibodies. In phage libraries, each clone represents a specific antibody-producing phage. On the basis of results obtained from phage-ELISA, 13, 15, 14, and 15 phage clones displaying scFv antibodies showed the highest signal to detect nTcdA, nTcdB, rTcdA, and rTcdB, respectively (Fig. 1C and Fig. S2 at https://figshare.com/s/54a86cbfe656e260a75f). Additionally, the ability of phages with the highest ELISA signals to detect native toxins was evaluated using scFv-ELISA, in which selected scFvs were used as primary antibodies. Based on the results, of 27 selected scFvs for nTcdA and rTcdA, 13 clones were able to detect nTcdA. Also, of 30 selected scFvs for nTcdB and rTcdB, 15 clones were able to detect nTcdB (Fig. 1D). Interestingly, 12 scFvs screened for rTcdA or rTcdB showed low binding affinity to nTcdA or nTcdB, despite high binding to the panning antigen.

### Characterization of specific scFv antibodies selected against TcdA or TcdB

The sequence analysis of scFvs with high binding activity revealed that the obtained sequences belonged to 18 unique scFvs, which all of them contained six CDRs and named according to the type of toxin and their corresponding numbers in 96-well microtiter plates, including five scFvs detecting nTcdA (A-A6, A-C9, A-C12, A-D7, A-D8), two scFvs detecting rTcdA (rA-A12, rA-C2), seven scFvs detecting nTcdB (B-B10, B-C12, B-E4, B-F2, B-F5, B-F11, B-H2) and four scFvs detecting rTcdB (rB-B4, rB-B5, rB-C4, rB-F3) (Fig. S3 at https://figshare.com/s/54a86cbfe656e260a75f). The sequence data of the selected scFvs were submitted to GenBank (Table S1 at https://figshare.com/s/54a86cbfe656e260a75f).

**Fig 2:**
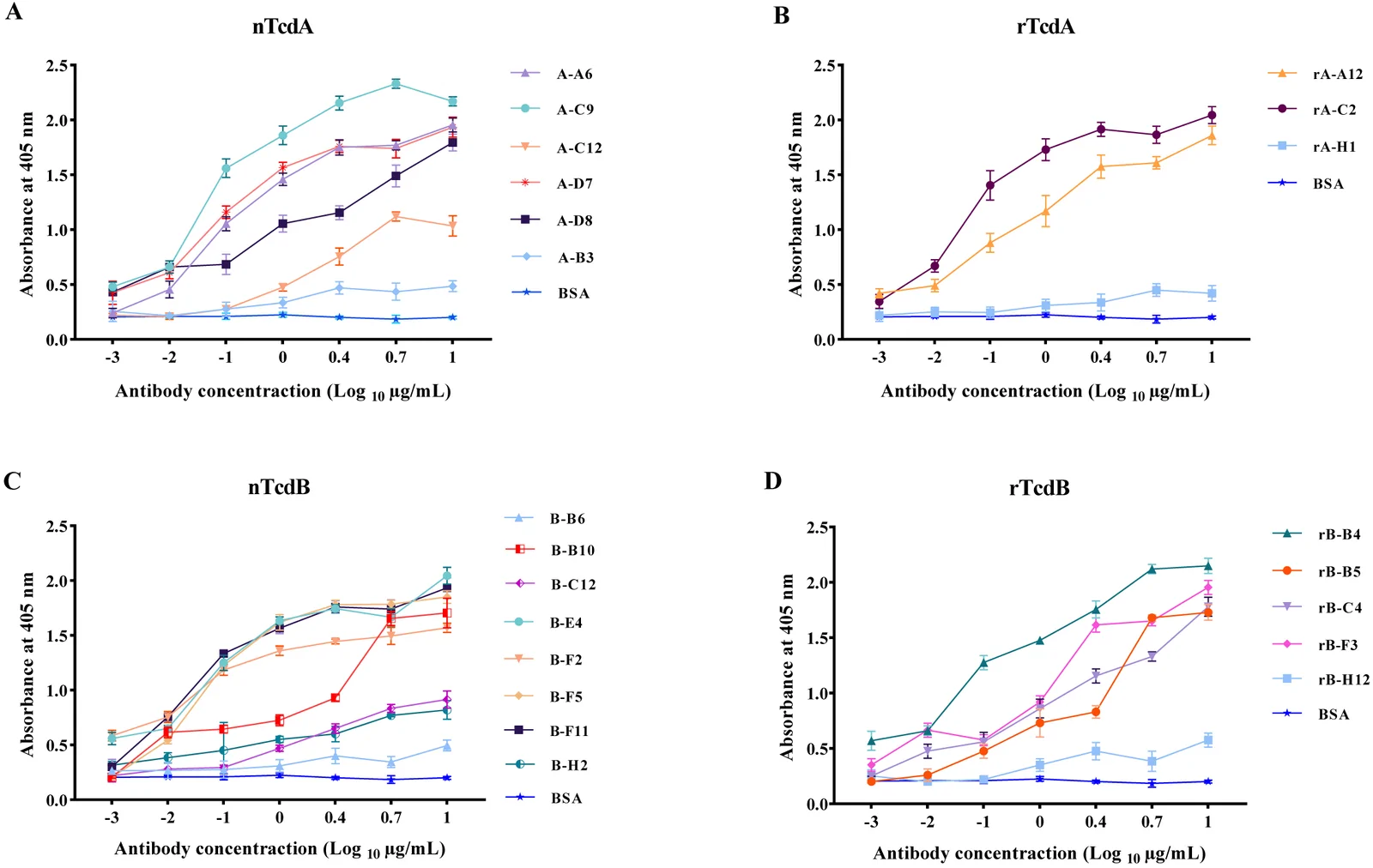
Titration-ELISA on native toxins for detecting binding activity of scFv antibodies selected for (**A**) native TcdA (nTcdA) from *C. difficile* RT084; (**B**) CROP domain fragment of TcdA (rTcdA) from *C. difficile* RT084; (**C**) native TcdB (nTcdB) from *C. difficile* RT017; and (**D**) CROP domain fragment of TcdB (rTcdB) from *C. difficile* RT017. BSA was used as negative control. Data shown are means ± SD of three independent experiments.

To better characterize the efficiency of specific scFv antibodies against native toxins, the binding activity of 18 selected scFvs (seven for TcdA and 11 for TcdB) was investigated by titration-ELISA using different concentrations of scFvs as primary antibodies, and nTcdA or nTcdB as antigens. A-B3 and rA-H1 antibodies isolated for TcdA, and B-B6 and rB-H12 antibodies isolated for TcdB that showed very low absorption in scFv-ELISA were used as negative control. The results showed that all scFvs exclusively detected their respective toxin in a concentration-dependent manner. Although the binding activity of some scFvs was weak at lower concentrations (0.001–0.1 µg/mL), six scFv antibodies for detecting TcdA, including A-A6, A-C9, A-D7, A-D8, rA-C2, and rA-A12 (Fig. 2A and B), and seven for detecting TcdB, including B-B10, B-E4, B-F2, B-F5, B-F11, rB-B4, and rB-F3 (Fig. 2C and D), showed strong binding activity in ELISA, and even at lower concentration (0.1 µg/mL). However, the binding activity of scFvs was greatly decreased (about ≤50% signal intensity) at the lowest concentration (0.01 µg/mL) compared to the highest concentration (10 µg/mL).

### Cell viability and toxin neutralization in Caco-2 cells

MTT [3-(4,5-dimethylthiazol-2-yl)2,5-diphenyltetrazolium bromide] assay was carried out to examine the cytotoxic effects of different concentrations of TcdA or TcdB for 4, 8, 24, and 48 h by measuring the reduction of tetrazolium salts to colored formazan products. Results showed that the viability of Caco-2 cells was significantly decreased after incubation with nTcdA or nTcdB in a concentration-dependent manner compared with untreated cells (*P* < 0.001). The concentration of 25 µg/mL of nTcdA led to 80% of cell death, whereas a greater efficiency for TcdB was observed for inducing cytotoxicity, so that a concentration of 10 µg/mL of TcdB could induce ~80% toxicity, representing a lethal dose of 80% (LD80) (Fig. S4 at https://figshare.com/s/54a86cbfe656e260a75f).

**Fig 3:**
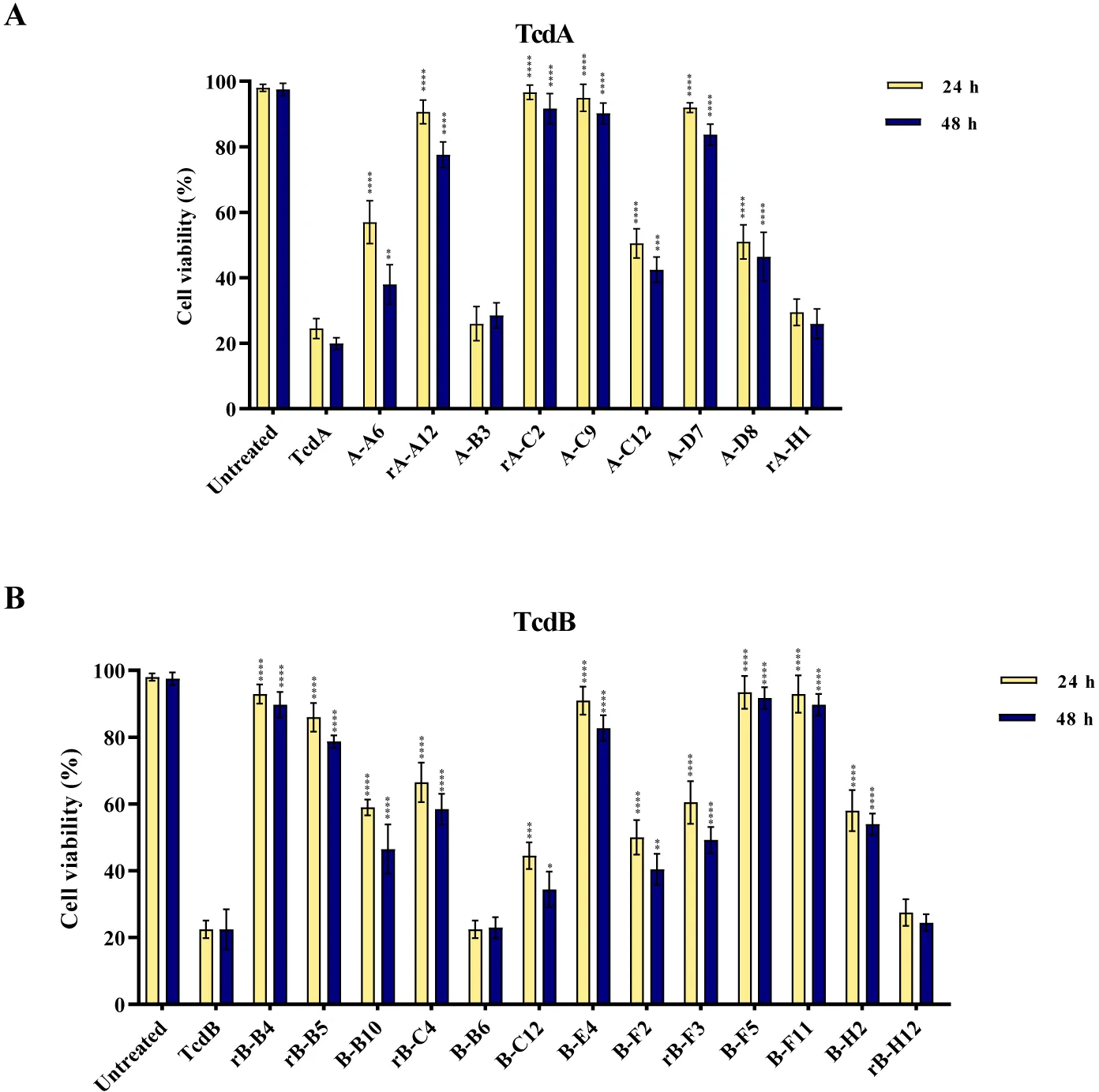
Cell viability of Caco-2 cells treated with (**A**) anti-TcdA scFv antibodies (1 µg/mL) and native TcdA (25 µg/mL) of *C. difficile* RT084; and (**B**) anti-TcdB scFv antibodies (1 µg/mL) and native TcdB (10 µg/mL) of *C. difficile* RT017 for 24 h at 37°C. Data shown are means ± SD of three independent experiments. A *P* value of <0.05 was considered as significant (*P < 0.05; **P < 0.01, ***P < 0.001, ****P < 0.0001) by unpaired Student’s *t* test and one-way ANOVA statistical analysis.

Interestingly, pre-incubation of toxins at LD80 with either A-A6, rA-A12, rA-C2, A-C9, A-C12, A-D7, or A-D8 for neutralization of TcdA, and rB-B4, rB-B5, B-B10, rB-C4, B-C12, B-E4, B-F2, rB-F3, B-F5, B-F11, or B-H2 for neutralization of TcdB increased cell viability compared with cells treated with toxin alone at time points 24 and 48 h (*P* < 0.0001) (Fig. 3). A-B3 and rA-H1 isolated for TcdA, and B-B6 and rB-H12 isolated for TcdB were used as negative control. A high level of toxin neutralization (70 to 100%) was achieved by rA-A12, rA-C2, A-C9, and A-D7 for neutralization of TcdA, and rB-B4, rB-B5, B-E4, B-F5, and B-F11 for neutralization of TcdB when toxins were used at LD80. Notably, five scFvs including rA-C2, A-C9, rB-B4, B-F5, and B-F11 were significantly more potent than other scFvs at both time points, and showed the highest level of neutralization (~90% at LD80).

### *In vitro* neutralization in Vero cells

**Fig 4:**
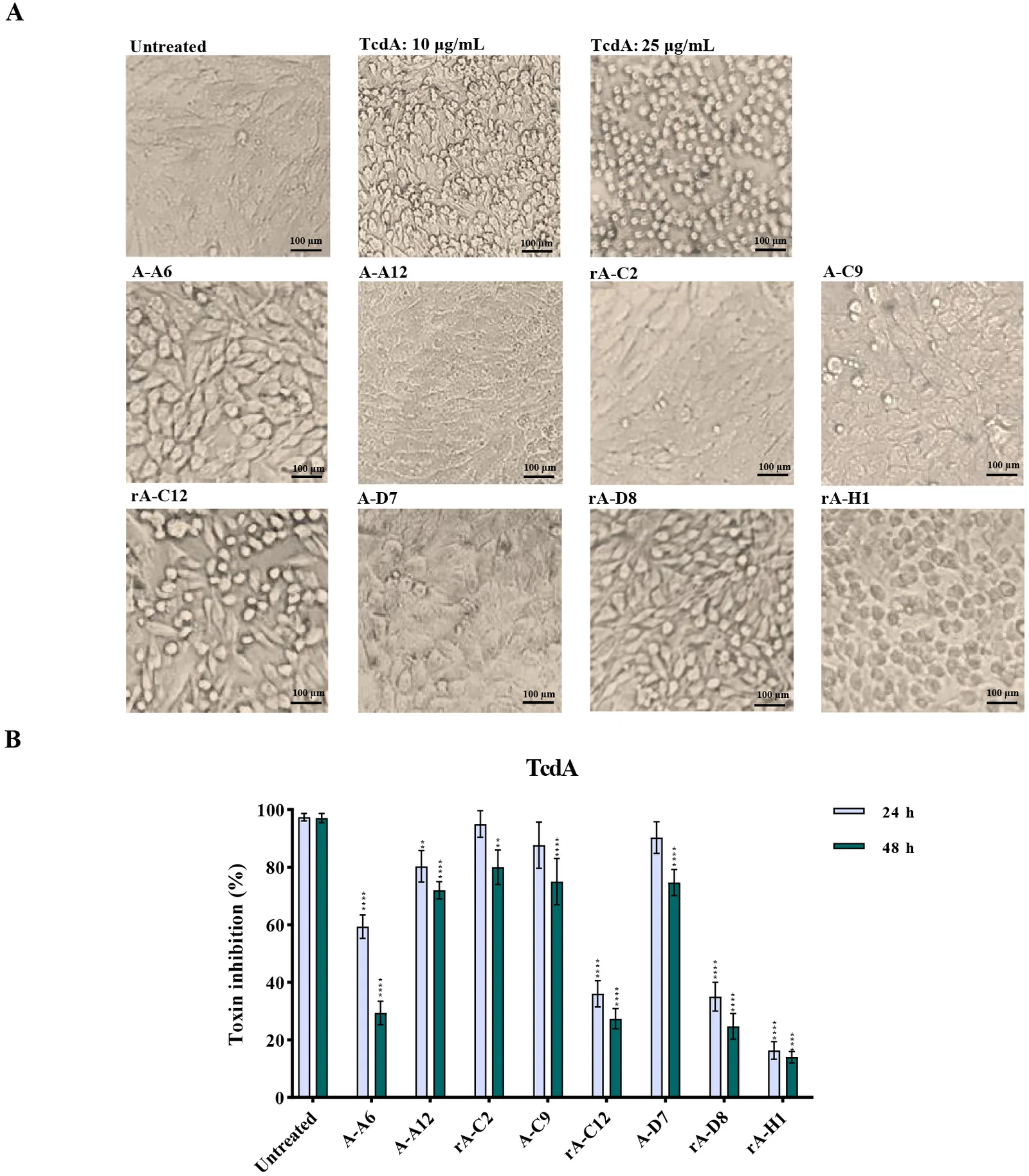
*In vitro* neutralization activity of anti-TcdA svFv antibodies (1 µg/mL) on cell rounding induced by native TcdA (25 µg/mL) from *C. difficile* RT084 on Vero cells. (**A**) Microscopic cell morphology of Vero cells co-treated with native TcdA, and specific anti-TcdA svFv antibodies for 24 h at 37°C in comparison with untreated Vero cells. Light microscopy (400x magnification), scale bar = 100 µm. (**B**) The percentage of inhibition of cell rounding in Vero cells treated with anti-TcdA svFv antibodies for 24 and 48 h at 37°C in comparison with untreated cells. The number of round cells was normalized to 100% cytotoxicity for TcdA. Data shown are means ± SD of three independent experiments. A *P* value of <0.05 was considered as significant (*P < 0.05; **P < 0.01, ***P < 0.001, ****P < 0.0001) by unpaired Student’s *t* test and one-way ANOVA statistical analysis.

Similarly, the neutralization activity of scFv antibodies on TcdA or TcdB at LD80 was tested on Vero cells. In the first phase of screening, the results of the morphological examination showed that TcdA and TcdB caused disruption of actin cytoskeleton, resulting in rounding up of Vero cells. Intriguingly, all tested scFvs somewhat reduced the percentage of round cells induced by nTcdA compared to control cells (the percentage of round cells in wells treated with nTcdA alone) (Fig. 4A). In more detail, the half maximal inhibitory concentration (IC50) values were obtained by pre-mixing of nTcdA with either rA-A12, rA-C2, A-C9, or A-D7 scFv antibodies (Fig. 4B). The highest *in vitro* neutralization activity of TcdA was achieved by rA-C2 and A-C9 (>80% reduction of cell rounding) at both time points 24 and 48 h. Likewise, the application of different concentrations of scFvs demonstrated that rA-C2 and A-C9 were more potent than other isolated scFvs, since 1 ng/mL of these scFvs was sufficient to reduce cell rounding induced by nTcdA to more than 50% (Fig. S5 at https://figshare.com/s/54a86cbfe656e260a75f).

**Fig 5:**
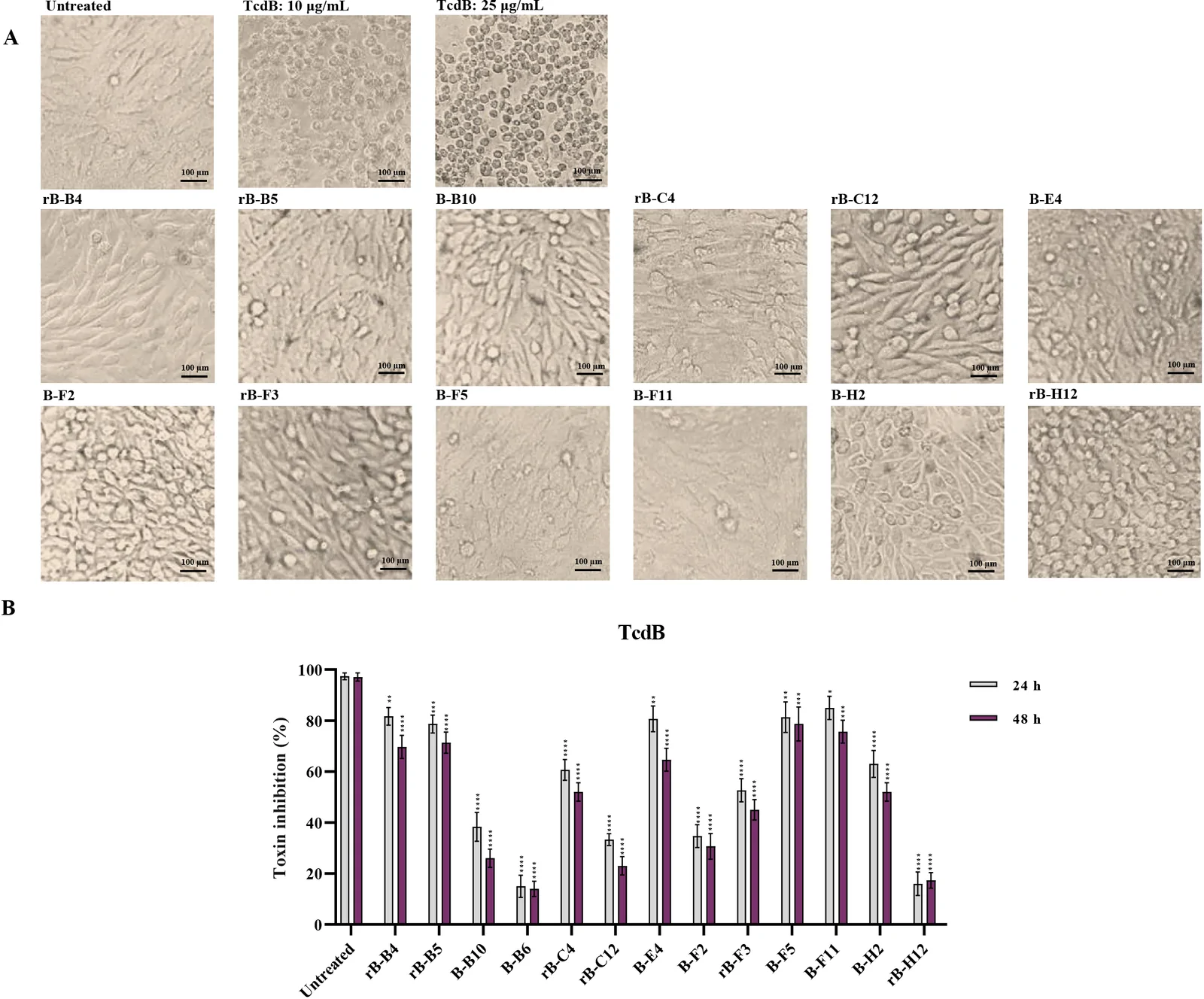
*In vitro* neutralization activity of anti-TcdB svFv antibodies (1 µg/mL) on cell rounding induced by native TcdB (10 µg/mL) from *C. difficile* RT017 on Vero cells. (**A**) Microscopic cell morphology of Vero cells co-treated with native TcdB, and specific anti-TcdB svFv antibodies for 24 h at 37°C in comparison with untreated Vero cells. Light microscopy (400x magnification), scale bar = 100 µm. (**B**) The percentage of inhibition of cell rounding in Vero cells treated with anti-TcdB svFv antibodies for 24 and 48 h at 37°C in comparison with untreated cells. The number of round cells were normalized to 100% cytotoxicity for TcdB. Data shown are means ± SD of three independent experiments. A *P* value of <0.05 was considered as significant (*P < 0.05; **P < 0.01, ***P < 0.001, ****P < 0.0001) by unpaired Student’s *t* test and one-way ANOVA statistical analysis.

Moreover, selected anti-TcdB scFvs were able to neutralize nTcdB, since pre-mixing of TcdB with either rB-B4, rB-B5, B-E4, rB-F3, B-F5, or B-F11 resulted in more than 50% toxin neutralization (Fig. 5A). In accordance with MTT assay, the highest *in vitro* toxin neutralization was achieved by rB-B4, rB-B5, B-F5, and B-F11 (>75% reduction of cell rounding) at both time points 24 and 48 h (Fig. 5B). These scFvs also showed a high potency of neutralizing in lower concentrations, as far as the application of 1 ng/mL of B-F5 and rB-B4 was capable of reducing round cells to more than 50% (Fig. S5 at https://figshare.com/s/54a86cbfe656e260a75f).

Intriguingly, treatment of Vero cells with specific anti-TcdA did not significantly alter cell rounding induced by TcdB compared with control, whereas treatment with anti-TcdB B-F5 antibody showed favorable binding with TcdA and significantly reduced cell rounding caused by TcdA >50% (Fig. S6 at https://figshare.com/s/54a86cbfe656e260a75f).

### Effects of scFv antibodies on the expression level of inflammation-related genes in Caco-2 cells treated with TcdA or TcdB

**Fig 6:**
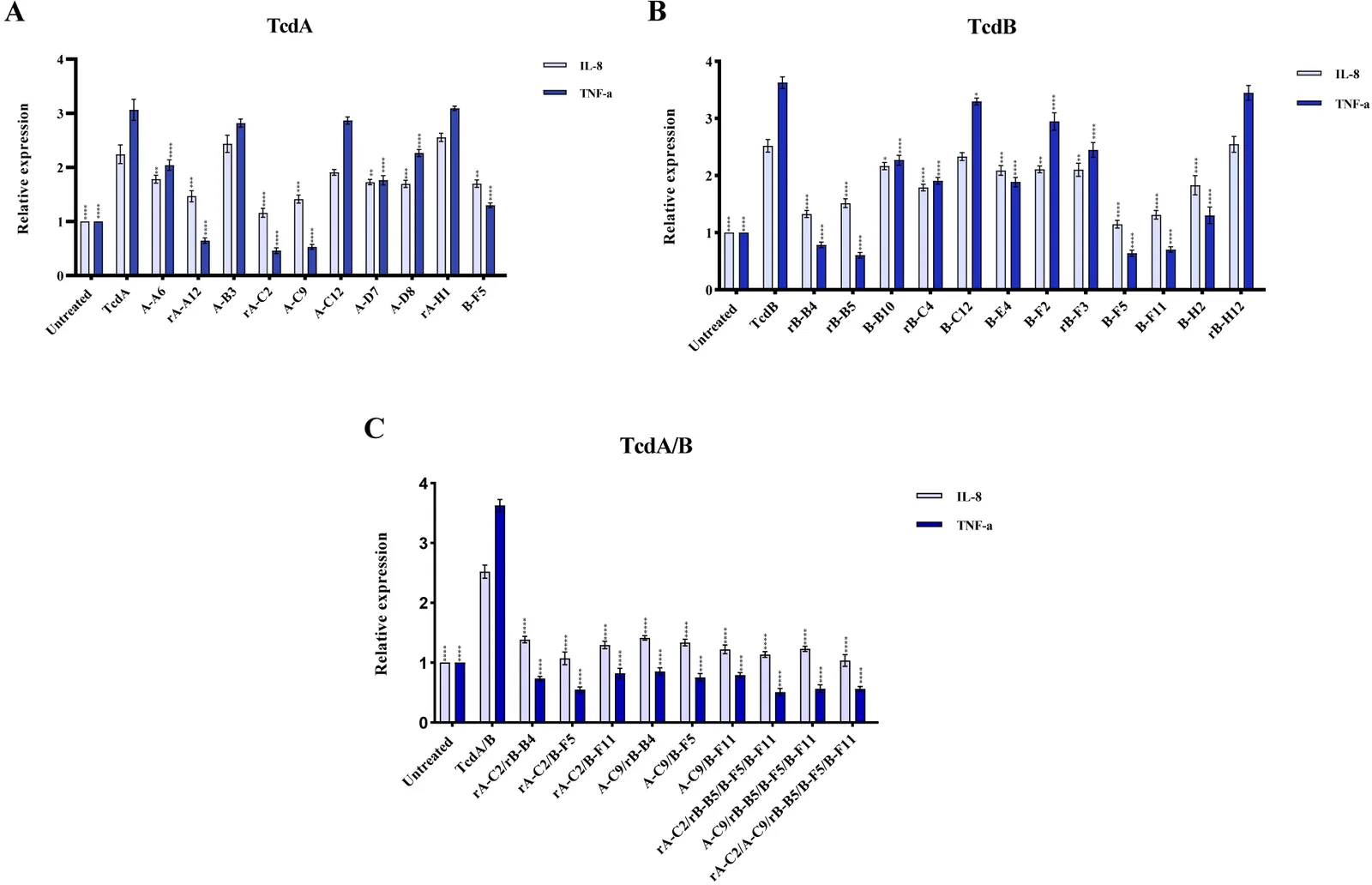
Relative expression of IL-8 and TNF-α genes in Caco-2 cells upon co-treatment with (**A**) anti-TcdA scFv antibodies (1 µg/mL) and native TcdA (10 µg/mL) of *C. difficile* RT084; (**B**) anti-TcdB scFv antibodies (1 µg/mL) and native TcdB (10 µg/mL) of *C. difficile* RT017; and (**C**) a mixture of anti-TcdA/anti-TcdB scFv antibodies (at final concentration 1 µg/mL) and combination of TcdA/B (10 µg/mL), which were measured by using RT-qPCR. Gene expression data were normalized to β-actin as the reference gene. Data were presented as mean ± SD from three independent experiments. A *P* value of <0.05 was considered significant (*P < 0.05; **P < 0.01) by unpaired Student’s *t* test and one-way ANOVA statistical analysis.

As previously documented, both TcdA and TcdB can induce the expression of the proinflammatory cytokines such as TNF-α and IL-8 in different human intestinal cell lines (5, 25). Hence, the RT-qPCR assay was used to examine the inhibitory effects of scFvs on the expression level of inflammation-related genes in Caco-2 cells stimulated by toxins. Based on the results, nTcdA and nTcdB significantly upregulated the expression level of TNF**-**α and IL-8 in Caco-2 cells (Fig. 6). Inversely, the treatment of Caco-2 cells with A-A6, rA-A12, rA-C2, A-C9, A-D7, A-D8, and B-F5 significantly modulated TcdA-induced TNF-α and IL-8 expression level compared to Caco-2 cells treated with nTcdA alone, whereas no effect was indicated for A-B3 and rA-H1 treatments that were used as negative control (Fig. 6A). Based on the results, the best neutralizing potency was observed for rA-C2 and A-C9 treatments, which showed the highest inhibition for the expression level of TNF-α and IL-8 in Caco-2 cells stimulated by 10 µg/mL nTcdA compared to the control (no antibody added). In consistent with the results obtained for toxin neutralization using Vero cells, treatment of Caco-2 cells with anti-TcdB B-F5 antibody significantly altered TcdA-induced TNF-α and IL-8 mRNA expression. Similarly, treatment of Caco-2 cells with anti-TcdB antibodies rB-B4, rB-B5, B-B10, rB-C4, B-E4, B-F2, B-F5, rB-F3, B-F11, and B-H2 significantly reduced TcdB-induced TNF-α and IL-8 expression, while negative controls (B-B6 and rB-H12) were not able to downregulate the expression level of these genes in the presence of 10 µg/mL TcdB (Fig. 6B). Moreover, the highest inhibitory effect was observed for rB-B4, B-F5, and B-F11. However, there was no significant difference in the mRNA expression of TNF-α and IL-8 between other scFv groups.

### Effects of a mixture of scFvs on modulating the gene expression of proinflammatory cytokines in Caco-2 cells treated with *C. difficile* toxins

It was proposed that antibodies binding to different epitopes without overlapping may exert significant synergic effects. Therefore, to obtain greater potencies for the neutralization activity of antibodies, the five scFvs showing the highest neutralizing activity (rA-C2, A-C9, rB-B4, B-F5, and B-F11) was selected and a combination of two to five scFvs at equal volume (final concentration: 1 µg/mL) was tested on the mixture of TcdA/B using an *in vitro* assay. Based on the results, the combination of anti-TcdA and anti-TcdB scFv antibodies showed a notable inhibiting effect on reducing TcdA/B-induced TNF-α and IL-8 expression at mRNA level in comparison to Caco-2 cells stimulated by toxins alone. However, no increases in potency were observed when a greater number of scFvs was mixed (Fig. 6C), as far neutralization activity of a combination of five scFvs (rA-C2, A-C9, rB-B4, B-F5, and B-F11) was not stronger than the combination of two specific scFvs (rA-C2 and B-F5).

### Homology model building and molecular docking

To identify epitopes involved in the interaction of scFvs having the highest neutralizing activity (rA-C2, A-C9, rB-B4, B-F5, and B-F11) with their respective toxin, molecular docking was performed using Haddock server service. Three-dimensional (3D) structural models of the selected scFvs were generated by I-TASSER server service and the total model quality was calculated using ProSA Z-score plot, implicating a high quality of models (Fig. S7 at https://figshare.com/s/54a86cbfe656e260a75f). Docking outputs revealed interaction between scFvs and TcdA (PDB ID: 7pog) or TcdB (PDB ID: 7n9y), showing a large binding interface primarily comprising CDRL3, CDRH3, and CDRH2 of scFv fragments (Table 2). In more detail, steric clashes between scFv and toxin were mostly restricted to CROP domain of TcdA or TcdB. In this regard, the antibody derived from CROP-TcdA, that is, rA-C2, interacted with LR3 of the CROP domain of TcdA (Fig. S8 at https://figshare.com/s/54a86cbfe656e260a75f). These binding regions are also found in interaction with actoxumab. A-C9 exhibited restoration of strong binding to CROP domain and the transmembrane domain (TLD) of TcdA, which is located in epitopes existing between 1410 and 2033 aa. In addition, we examined the binding affinity of B-F5 to TcdA. The binding ability of B-F5 with TcdA was slightly weakened in comparison with other scFvs. The analysis of B-F5-TcdA interaction revealed that B-F5 can detect epitopes existing in the CROP domain of TcdA. Accordingly, the interactions of three anti-TcdB antibodies (B-F5, B-F11, rB-B4) were restricted to epitopes between 1810 and 2024 aa of the CROP domain of TcdB (Fig. S9 at https://figshare.com/s/54a86cbfe656e260a75f). Interestingly, rB-B4 resulted in similar interaction to bezelotoximab, which may explain the molecular mechanism underlying the restoration of rB-B4 binding to TcdB.

**TABLE 2:** Amino acid residues of anti-TcdA and anti-TcdB scFv antibodies involved in the hydrogen bonds with the antigens^*a*^

	Position					
	Heavy chain			Light chain		
	CDRH1	CDRH2	CDRH3	CDRL1	CDRL2	CDRL3
rA-C2	Ser: 33	Gln: 54, Ser: 55	Asp: 101, Arg: 104	Ser: 162, Ser: 164, Tyr: 166	Tyr: 183, His: 184, Ile: 187, Leu: 188, Gln: 189	Ser: 225, Asn: 230
A-C9	-	Asn: 54, Arg: 58, Arg: 61	Gly: 102, Ser: 103	Ser: 160, Gln: 161, Ser: 162, Ser: 164	Arg: 184	Thr: 203
rB-B4	Ser: 33	Gly: 55, Arg: 56, Arg: 59	-	Ser: 164, Ser: 165, Tyr: 166	Ser: 186, Ser: 187	Tyr: 226
B-F5	Thr: 30, Tyr: 34, Ala: 35	Asn: 54, Ars: 79	Lys: 100, Arg: 102	Gln: 161, Ser: 162, Tyr: 166	Tyr: 183, Arg: 184	Arg: 226
B-F11	Tyr: 34	Arg: 56, Arg: 58	Tyr: 102, Gln: 103	-	Ala: 185, Ser: 186, Ser: 187	Tyr: 226, Thr: 228

^*a*^ CDR: complementarity determining regions; H: heavy chain; L: light chain.

## DISCUSSION

Toxigenic *C. difficile* strains can disrupt the colonic epithelium leading to intestinal inflammation and tissue damage, which are considered as major characteristics of CDI (1, 26, 27). The initial damage in the gut is caused by TcdA, which provides deeper access for TcdB to underlying tissues, and leads to augmented inflammatory responses (28, 29). Therefore, the neutralization of TcdA and TcdB can interfere with the mechanism of *C. difficile* pathogenesis, resulting in improved clinical outcomes and more-durable therapeutic effects, such as reduction in symptom severity, recurrence rate, and death (17, 30, 31). So far, many studies have been conducted to examine the production of potent antibody-based neutralizing factors; however, bezlotoxumab is the only FDA-approved antibody for preventing rCDI yet (17). Although the application of bezlotoxumab has shown promising results for rCDI treatment due to reducing the relapse rate up to 40%, it has not been approved for acute CDI treatment (17, 32). Thus, development of new potent toxin-neutralizing antibodies remains crucial for CDI treatment. To date, phage display technology represents a potent *in vitro* selection technique for developing target-specific antibodies without need for experimental animals (12, 24). In this regard, the present study was aimed to identify and characterize a panel of novel human scFv antibodies targeting *C. difficile* TcdA and TcdB using phage display technique. To do this, a total of four antibody selections were performed through three rounds of biopanning using nTcdA or nTcdB. Additionally, several studies have demonstrated that the best neutralizing antibodies can target the CROP domain of toxins (16, 20, 33); therefore, recombinant CROP domains of each toxin, that is, rTcdA and rTcdB, were also used as antigens to isolate antibodies that specifically block these domains. Our results showed that three rounds of biopanning led to the selection of about 28 and 30 individual clones that, respectively, reacted with TcdA and TcdB in phage-ELISA, demonstrating that biopanning was successfully implemented (20, 34). Moreover, an alternating panning round was conducted on scFv fragments to more precisely characterize the selected clones and focus on particular epitopes related to cognate antigens (TcdA or TcdB) (35). This selection strategy gives a more native conformation of antigen-antibody binding and provides a higher number of available epitopes for scFv binding compared to phage selection, leading to isolating antibodies with higher affinity (34, 36). Interestingly, based on results obtained from scFv-ELISA, a drastically reduced binding activity was observed for five clones detecting nTcdA and seven antibodies detecting nTcdB compared to phage-ELISA. Since most of these antibodies were selected based on the recombinant domain of CROPs, it seems that binding regions of these antibodies are only available in the folding of recombinant antigens and do not exist in the 3D structure of nTcdA or nTcdB (16). Sequencing of clones with the highest absorption in ELISA led to the isolation of 18 unique scFv antibodies. Some of these scFvs showed higher binding activity even at very low concentrations (>0.01 µg/mL). Previous studies have also demonstrated a high binding activity for scFv antibodies to detect different antigens (22, 34, 36); however, there are few reports about the application of scFv antibodies for detecting *C. difficile* toxins. In this regard, Deng et al. reported the isolation of TcdB-neutralizing scFvs using phage display technology; however, the work did not progress beyond binding assays (37). Recently, Fühner et al. isolated antibodies targeting different domains of TcdB having the ability to detect TcdB at low concentration (1 nM) (16).

Both TcdA and TcdB toxins contain four domains, which can be considered for therapeutic purposes. The glucosyltransferase domain (GTD), TLD, or CROPs domains of TcdA and TcdB all harbor epitopes that can mediate binding of toxins to cell surface receptors, enzymatic activity, internalization of toxins, and cytotoxicity (16, 38 – 40). In this work, a total of 18 scFv antibodies were tested to evaluate their neutralizing capability using *in vitro* assays. As reported in previous studies, various cell lines can show different sensitivity to TcdA and TcdB (4, 29); thus, two cell lines, Caco-2 and Vero cells, were used in the present work to assess the potential neutralizing activity of scFv antibodies. To that end, all 18 mAbs increased the cell viability of Caco2 cells and decreased the percentage of cell rounding of Vero cells treated with TcdA or TcdB, however, showed dissimilar potency and therefore distinct neutralizing effects. However, five (rdA-C2, A-C9, rB-B4, B-F5, B-F11) had neutralization efficacy of more than 75% and showed neutralizing activity ranging from 10 to 0.01 µg/mL against TcdA and TcdB at LD80. Moreover, the scFvs including A-A6, A-D8, B-B10, and B-F2 exhibited high binding activity to detect their respective toxin even at low concentrations (0.01 µg/mL), although had low efficacy to neutralize toxin probably due to harboring non-neutralizing epitopes. Neutralizing antibodies can simultaneously bind to antigens and block their function, while non-neutralizing antibodies can only bind to antigens (41, 42). Similarly, several studies have demonstrated that anti-toxin antibodies can contain neutralizing and non-neutralizing epitopes with different potency for neutralization (16, 42). In agreement with our findings, the results of other studies have also indicated that neutralizing antibodies can exert potent inhibitory activity against toxins even at low concentration (0.001 µg/mL) (16, 20). Another observation to note from this work is that B-F5, which was selected directly against TcdB, showed considerable neutralization efficacy for TcdA in cytotoxicity assays. Based on the homology of CROP regions of TcdA and TcdB, cross-reactive epitopes may exist for these toxins (4, 40) that may affect cell binding.

*In vitro* and *in vivo* studies have documented that anti-toxin antibodies can also modulate the inflammatory activity of TcdA and TcdB, thus inhibiting downstream signaling events that may lead to tissue damage and cell death (13, 25). This evidence led us to hypothesize that the selected scFvs may alleviate the proinflammatory responses activated by TcdA and TcdB in the colon. Our results demonstrated that 1 µg/mL scFvs is sufficient to block TcdA- or TcdB-mediated TNF-α and IL-8 expression in Caco-2 cells. Analyzing this data confirmed our earlier findings that anti-TcdA and anti-TcdB scFvs (rdA-C2, A-C9, rB-B4, B-F5, B-F11) can show high potency to inhibit cytotoxicity induced by toxins on cell monolayers. Accordingly, the combination of the scFvs isolated against TcdA and TcdB could lead to targeting different domains of toxins and improving neutralization and synergistic effects (13, 14, 43). Similarly, in our study, combinations of anti-TcdB scFvs with either rA-C2 or A-C9 scFvs in pairs exerted 100% toxin neutralizing; however, no increases in potency were observed when a greater number of scFvs were mixed. These scFvs already showed the best neutralization effect among other binders in the initial screening as single antibodies. Consistent with our findings, Andersen et al. isolated several specific variable heavy domain of heavy-chain (VHHs) antibodies binding to the CROP domain of TcdB that each was able to neutralize TcdB alone (25). Several studies exhibited that mAbs neutralizing the C-terminal domain of TcdB can inhibit the destructive effects of toxins and represent effective protection in *in vivo* assays (14, 33, 38, 43). Additionally, the neutralization mechanism of bezlotoxumab and actoxumab is through the blocking of the interaction of CROPs with cell surface receptors, which leads to inhibition of cell binding and interaction of toxins with CSPG4 receptor (44, 45). Based on our molecular docking results, some of the most potent scFvs isolated in this study can neutralize toxins in a similar manner with bezlotoxumab or actoxumab. The most-potent neutralizing antibody for TcdA (rA-C2) binds to repeating epitopes within LR3 of the CROP domain of TcdA. Similarly, this epitope was previously reported to be utilized by actoxumab for neutralizing TcdA (46). Nevertheless, A-C9 binds to specific receptor-binding sites within TLD, which plays a key role in toxin internalization and prevents toxin internalization (13, 47). The interaction of anti-TcdB antibodies (rB-B4, B-F5, and B-F11) was restricted to CROP domain of TcdB. Interestingly, rB-B4 demonstrated similar interaction as bezelotoximab, which may explain the molecular mechanism underlying the restoration of rB-B4 binding to TcdB (44). Analysis of the interaction of B-F5-TcdA revealed that B-F5 can detect epitopes existing in the CROP domain of TcdA. As mentioned earlier, assessment of the homology of the CROP regions of TcdA and TcdB showed cross-reactive epitopes in these regions (44), hence, B-F5 can bind to repeating CROP domain epitopes of both toxins; however, its neutralization efficacy was higher for TcdB than TcdA.

**Fig 7:**
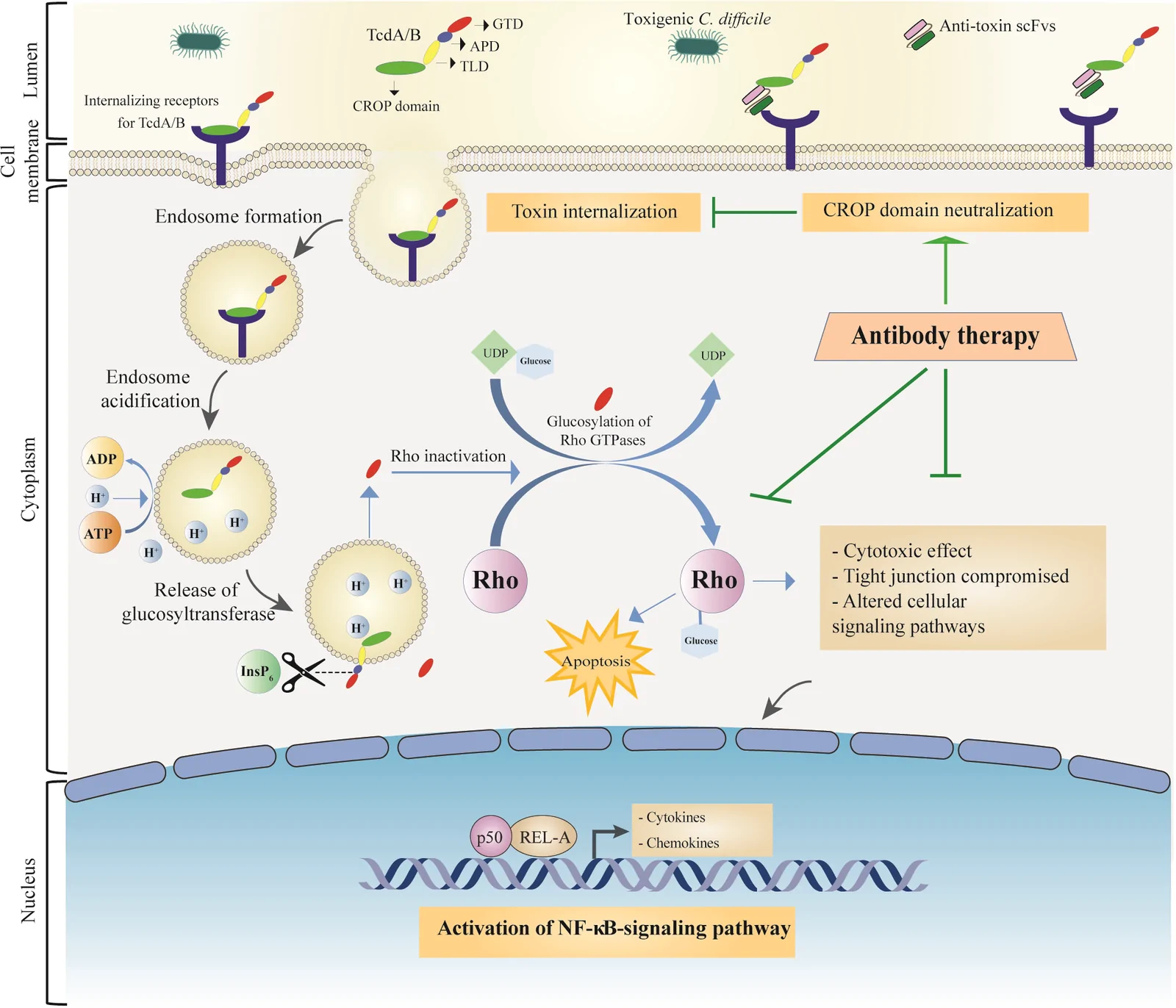
Schematic overview of the application of antibody for the treatment of CDI. In general, both toxins consist of the glucosyltransferase domain (GTD), the auto-protease domain (APD), the transmembrane domain (TLD), and the combined repetitive oligopeptides domain (CROP). Both toxins can interact with their specific receptors using CROP domain. Following receptor binding, the toxin-receptor complex is endocytosed and toxins internalize into the intestinal epithelial cells (IECs). The influx of protons leads to the acidification of endosomes; in this condition, GTD and APD domains are translocated through transmembrane pore formation. Inositol hexakisphosphate (InsP6) activates APD and releases GTD. The GTD glycosylates inactivate Rho proteins (and probably other related GTPases), resulting in downstream cellular changes such as destruction of cytoskeleton, disruption of tight junctions, production of inflammatory cytokines in lymphocytes, macrophages, and dendritic cells, and apoptosis induction. These outcomes can lead to neutrophil influx, causing pseudomembrane colitis formation. The application of antibodies selected against the CROP domain of TcdA or TcdB can inhibit toxin internalization, which helps modulate the destructive effects of toxins on epithelial cells and ameliorate homeostatic immune responses.

In conclusion, we successfully applied phage display technique for the isolation of a panel of novel fully human monoclonal antibodies targeting TcdA and TcdB of *C. difficile*. The best toxin neutralizers were rA-C2, A-C9, rB-B4, B-F5, and B-F11, which showed a higher potency in different *in vitro* assays, including ELISA and toxin neutralization. Based on our results, at least two neutralization mechanisms including inhibition of cell surface binding of toxins and inhibition of toxin internalization can be proposed for the isolated scFvs in this work. Therefore, these antibodies can affect downstream signaling pathways involved in the inflammation and downregulation of the gene expression of proinflammatory cytokines, which contribute to multiple cell processes crucial to disease progression such as apoptosis (Fig. 7). The present research provided more evidences for *C. difficile* toxin neutralization at *in vitro* level, thus further research using proper animal models and clinical trials are required to evaluate safety, affinity, efficacy, and pharmacokinetic profile of these scFvs *in vivo*. Moreover, the toxin-neutralizing ability of these scFvs should be assessed using a larger cohort of *C. difficile* strains in clinical setting. Taken together, such scFvs potentially can be considered as promising tools for developing new therapeutic agents against CDI.

## MATERIALS AND METHODS

### *C. difficile* strains and culture conditions

In this study, we used TcdA-positive/TcdB-negative *C. difficile* RT084 (A^+^B^-^) and TcdA-negative/TcdB-positive *C. difficile* RT017 (A^-^B^+^), which were previously characterized in the Department of Anaerobic Bacteriology at Research Institute for Gastroenterology and Liver Diseases in Tehran, Iran (48). The strains were cultured on cycloserine-cefoxitin-fructose agar (CCFA, Mast, UK) supplemented with 5% (vol/vol) sheep blood under anaerobic conditions (85% N_2_, 10% CO_2_, and 5% H_2_) (Anoxomat Gas Exchange System, Mart Microbiology BV, Lichtenvoorde, Netherlands) at 37°C for 48–72 h, and then used to prepare a bacterial suspension equal to 2 McFarland turbidity standard in 0.85% sterile saline.

### Native toxin purification and toxin subdomains expression

The toxin purification was carried out as described previously with slight modifications (49). In brief, the suspension (100 µL) of *C. difficile* strains RT084 (A^+^B^-^) and RT017 (A^-^B^+^) were prepared and transferred to a 10 mL pre-reduced brain heart infusion (BHI) broth and incubated at 37°C under agitation at 120 rpm for 72 h. After that, cells and debris were removed by centrifugation at 8,000 × g, 4°C for 15 min. The supernatant was aseptically filtered through a membrane filter with 0.22 µm pore size, and stored at −80°C until analysis. This supernatant was inoculated with phosphate-buffered saline (PBS: 5.85 g/L NaCl, 4.72 g/L Na_2_HPO_4_, 2.64 g/L NaH_2_PO_4_·2H_2_O, pH = 7.2) in a dialysis bag (12–14 kDa exclusion limit; Sigma-Aldrich, Germany) to remove the media and low molecular weight proteins. The presence of TcdA or TcdB toxins was exanimated using ELISA (Generic Assays, Germany) according to the manufacturer’s instructions.

To prepare recombinant proteins, DNA fragments encoding CROP domain of TcdA (1868–2704 aa) of *C. difficile* RT084, and CROP domain of TcdB (1802–2366 aa) of *C. difficile* RT017 were amplified using primers incorporating restriction endonuclease sites of *Nco1* and *XhoI* (Table S2 at https://figshare.com/s/54a86cbfe656e260a75f).

Amplified fragments were restricted by their respective endonuclease sites, sub-cloned into pET28a (+) bacterial expression vector (Cat. no. 69864-3; Novagen, USA) containing a kanamycin resistance cassette and a C-terminal 6× His-tagged, and transformed into the Rosetta (DE3) strain of *E. coli*. Protein expression was induced at OD600 = 0.4–0.5 with 1 mM isopropyl-β-D-thiogalactoside (IPTG) for 16 h at 30°C. The recombinant TcdA (rTcdA) and TcdB (rTcdB) were purified under native conditions using IMAC (Thermo Fisher Scientific, USA), as per the manufacturer’s instructions. The purified proteins were stored at −80°C in 1× PBS buffer (pH = 7.2). The expression of proteins was estimated by separation in 12% SDS-PAGE and confirmed using western blotting with anti-His tag antibody conjugated with horseradish peroxidase (HRP) (Abcam, UK) (50). Protein concentration was determined using BCA protein assay kit (DNAbiotech, Tehran, Iran). A schematic overview of the method of preparation of native and recombinant toxins is presented in Fig. S10 at https://figshare.com/s/54a86cbfe656e260a75f.

### Enrichment and screening of the phage display library

The Tomlinson I and J naïve scFv phage library was pooled and grown in 2× YT medium (16 g/L tryptone, 10 g/L yeast extract, 5 g/L NaCl, pH = 7.2) containing 100 µg/mL ampicillin, 50 µg/mL kanamycin, and 1% (wt/vol) glucose upon infection with M13K07 helper phage suspension (about 10^9^ pfu/mL). Phages were precipitated with PEG/NaCl (20% PEG 6000 and 2.5 M NaCl) followed by centrifugation at 3,300 × g and then resuspended in 1 mL of 1× PBS buffer. After determining the phage titer by plaque assay, library biopanning was carried out as described previously with some modifications (51).

In brief, the scFv phages from Tomlinson I and J library were mixed and the phage suspension (~10^13^ pfu/mL) was first panned with 2% (wt/vol) BSA (Fluka, Neu-Ulm, Germany) in 1× PBS buffer at 25°C for 4 h to remove non-specific phages. To begin screening, high-binding flat-bottom polystyrene microplates (Sigma-Aldrich, Germany) were coated with ~100 µg/mL solution of each antigen (nTcdA, nTcdB, rTcdA, and rTcdB) in PBS buffer, stored at 4°C for 16 h, and then blocked with 3% (wt/vol) BSA in 1× PBS. The plate coated with antigen was exposed to the phage supernatant and incubated for 2 h at 25°C. At the end of incubation times, the microtiter plates were washed more than 20 times with PBS containing 0.1% Tween 20 (PBST) to eliminate non-specific or low-affinity phages. The phages bound to antigen were eluted with trypsin-PBS solution (200 µL of 10 mg/mL trypsin stock solution in 10 mL PBS), reamplified through infecting *E. coli* TG1 cells (OD600 = 0.4), and used for the next round of biopanning. In each subsequent round, the concentration of antigens was reduced and washing conditions were more stringent. After three rounds of biopanning, the recovery rate of each round was calculated using the formula “Recovery rate = output phages/input phages.”

### Specificity assessment of the phage clones to TcdA and TcdB

Polyclonal phage-ELISA was carried out for each round of biopanning to determine enrichment of antigen-specific phages as described previously (34). In brief, 10 µg/mL of each antigen (nTcdA, nTcdB, rTcdA, rTcdA) was immobilized on microplate wells. After blocking with 3% skimmed milk powder (Fluka, Germany) in 1× PBS, the eluted phages obtained from each round were added to microplate wells and incubated for 2 h at 37°C. After three times of washing with PBST, an HRP-conjugated anti-M13-polyclonal antibody was added, and plates were incubated for 1 h at 37°C. The colorimetric reaction was determined in the presence of 3′,3′,5,5′-tetramethylbenzidine (TMB) substrate (Fermentase, Vilnius, Lithuania) after further washing. The reaction was stopped by adding 100 µL 1 N H_2_SO_4_, and the absorbance at 405 nm was determined using an ELISA reader (TECAN Sunrise, 450 nm, reference 620 nm). Reactions were considered positive when the corresponding ELISA readings were at least twice those of the wells containing 3% BSA as a negative control.

### Screening of specific monoclonal phages to TcdA and TcdB

To isolate monoclonal binders for toxins, phages were further screened in phage-ELISA as described previously (34). Briefly, the eluted phages obtained from the third round of biopanning were transformed into *E. coli* TG1 cells. Bacterial cells containing phage clones were plated on TYE medium (5 g/L NaCl, 5 g/L yeast extract, 10 g/L tryptone, pH = 7.2) supplemented with 100 µg/mL ampicillin and 1% (wt/vol) glucose. Single *E. coli* colonies derived from the biopanning (94 colonies) were cultured in a 96-well microtiter plate containing 2× YT supplemented with 100 µg/mL ampicillin and 1% (wt/vol) glucose. Individual monoclonal phage displaying the antibody fragment was released from bacterial cells via the infection of the helper phage (about 10^9^ pfu/mL) and used for screening ELISA as described previously.

### Identification of specific soluble scFv fragments to TcdA and TcdB

Screening by soluble scFv*-*ELISA was performed as a supplementary round for selecting antibodies with high specificity as described previously (34). In brief, monoclonal phages were transformed into *E. coli* HB2151 cells. Transformed bacteria were cultured in 2× YT medium containing 100 µg/mL ampicillin and 0.1% (wt/vol) glucose, induced with 1 mM IPTG at OD600 = 0.7–0.8, and then incubated at 30°C overnight with shaking at 200 rpm. After incubation, the cells were centrifuged (2,000 × g, 10 min, 4°C) and the individual supernatant containing the C-terminal c-myc tag-fused scFv antibody was used as the primary antibody to detect nTcdA and nTcdB by using ELISA. The binding specificity of scFvs was assessed with an anti-c-myc tag antibody (1:5,000) (Abcam, USA), followed by HRP-conjugated goat anti-mouse IgG (1:7,000) (Abcam, UK). The colorimetric reaction was detected as described previously.

### Characterization of scFv antibodies

Positive clones producing the highest absorbance (450 nm) were selected for DNA Sanger sequencing (Pishgam Biotech Co., Tehran, Iran) using pHEN primers (Table S2 at https://figshare.com/s/54a86cbfe656e260a75f). The IMGT/V-QUEST alignment tool (http://www.imgt.org) and BioEdit version 7.2.5 (Ibis Therapeutics, Carlsbad, CA, USA) were used for sequence alignment analysis (52). The positive phagemids were transformed to the SHuffle strain of *E. coli* and expression of scFvs was done using 1 mM IPTG, and then purified under native conditions by IMAC. Purification of scFv fragments was analyzed by SDS-PAGE and probed by western blotting using HRP-conjugated anti-His tag antibody (Abcam, UK). Protein concentration was measured using BCA protein assay kit. Additionally, titration-ELISA was performed according to the ELISA method described earlier. A dilution series of the scFv antibodies (0.001, 0.01, 0.1, 1, 2.5, 5, 10 µg/mL) was used for the detection of nTcdA and nTcdB (10 µg/mL). The lowest concentration of scFvs that could detect the antigens was considered as a working concentration.

### Cell culture and growth conditions

The Caco-2 (human colon adenocarcinoma cell line) and Vero cell lines (an African green monkey kidney cell line) were obtained from the Iranian Biological Resource Center (IBRC, Tehran, Iran) and cultured in high-glucose Dulbecco’s modified Eagle’s medium (H-DMEM) (Gibco, USA) supplemented with 1% (vol/vol) of non-essential amino acid (NEAA) (Gibco, USA), 10% (vol/vol) fetal bovine serum (FBS) (Gibco, USA), and 1% (vol/vol) penicillin/streptomycin (Sigma-Aldrich, Germany), and incubated at 37°C in 5% CO_2_ conditions.

### Cell viability assay

The working concentration of TcdA and TcdB that provided ~90% toxicity was determined by a colorimetric assay based on the cleavage of the yellow tetrazolium salt MTT (Sigma-Aldrich, Germany). To do this, various concentrations of nTcdA or nTcdB (5, 10, 25, 50, 100 µg/mL) were prepared for obtaining lethal dose (LD) of each toxin. After 4, 8, 24, and 48 h, the cells were washed twice with PBS (pH = 7.4) and then 90 µL of culture medium and 10 µL/well of MTT (5 mg/mL in PBS) were added to each well. After incubation of cells for 4 h at 37°C in 5% CO_2_ conditions, 200 µL of dimethyl sulfoxide (DMSO) was added to each well for 15 min to stop the reaction. Untreated monolayers were used as negative controls and wells without cells served as blanks. The percentage of cell viability was calculated as follows: Cell viability (%) = (absorbance of treated cells × 100%)/absorbance of untreated cells.

For neutralization assay, selected scFv antibodies at concentration of 1 µg/mL were premixed with TcdA or TcdB (used at LD80) for 1 h at room temperature. The scFv-toxin mixture was transferred to Caco-2 cells that were seeded at 5 × 10^3^ cells/well in 96-well plates and incubated for 24 and 48 h at 37°C in 5% CO_2_. At the end of incubation times, medium was aspirated from all wells of the assay plates and the inhibitory effect of scFvs was calculated using the MTT method. All experiments were performed in triplicate.

### *In vitro* cytotoxicity assay

The cytotoxic activity of toxins was evaluated using Vero cells by breakdown of the actin cytoskeleton, which leads to cell rounding, as previously described (53). Briefly, Vero cells were seeded in a 96-well plate at 10^4^ cells/well. Two concentrations of nTcdA and nTcdB (10 and 25 µg/mL) were added to Vero cells and incubated for 24 and 48 h at 37°C in 5% CO_2_ conditions. The cytotoxic activity was determined by 90% cell rounding using an inverted microscope (Olympus Corporation, Tokyo, Japan) at 400× magnification. Cell images were taken and the percentage of round cells was determined by ImageJ software-assisted counting.

For neutralization assay, each antibody was premixed at the indicated concentrations (1 µg/mL) with nTcdA or nTcdB at consecrations 250 and 100 µg/mL, respectively, for 1 h at room temperature. After incubation, the scFv-toxin mixtures were added to the cells. Cell images were taken and the percentage of round cells in different treatments was determined by ImageJ, and then normalized to the percentage of round cells in the wells treated with nTcdA or nTcdB. Additionally, the efficiency of different concentrations of scFvs (0.00001–10 μg/mL) for toxin neutralization was determined as described previously. All experiments were performed in triplicate.

### Treatment of Caco-2 cells with toxins and specific scFv antibodies

To evaluate the effects of selected scFvs to modulate inflammatory responses induced by toxins, Caco-2 cells were seeded into 24-well plates at a density of 2.5 × 10^4^ cells*/*well. Cell treatments were carried out by premixing antibody (1 µg/mL) with nTcdA and/or nTcdB with the final concentration of 100 µg/mL to the wells and incubating for 24 h at 37°C in 5% CO_2_ conditions. To determine the efficiency of antibody combinations, all antibody mixtures were used in equal molar ratios with the final concentrations of 1 µg/mL. Untreated cells and Tox-S (100 µg/mL) without the addition of antibodies were used as controls. After the incubation time, the cells were lysed for RNA extraction and gene expression analysis. All treatments were run in triplicate.

### Total RNA isolation and cDNA synthesis

The treated Caco-2 cells were collected by centrifugation (800 × g, 10 min*,* 4*°*C) and used for RNA extraction, according to the manufacturer’s protocol of RNeasy Mini Kit (Qiagen, Germany). RNA purity was assessed by calculation of the ratio between absorbance at 260 and 280 nm (A260/A280) using a NanoDrop spectrophotometer (ND-1000, Thermo Scientific, USA). The isolated RNAs were stored at −80°C and then used for cDNA synthesis. Purified RNA was reverse transcribed to cDNA by the PrimeScript RT Reagent Kit (Takara, Japan) according to manufacturers' protocols. All cDNAs were kept at −20°C until further analysis.

### Quantitative real-time PCR

The mRNA expression level of interleukin-8 (IL-8) and tumor necrosis factor alpha (TNF-α) in Caco-2 cells treated with antibodies and Tox-S for 24 h were measured using a highly sensitive assay based on quantitative real-time PCR (RT-qPCR). Gene expression was evaluated by the Rotor-Gene Q (Qiagen, Germany) real-time PCR system using BioFACT 2X Real-Time PCR SYBR Green Master Mix (BIOFACT, South Korea). Oligonucleotide primers specific to each gene and their amplification conditions are listed in Table S2 at https://figshare.com/s/54a86cbfe656e260a75f. The β-actin housekeeping gene served as the reference gene. To confirm amplification specificity, a melting curve analysis and subsequent agarose gel electrophoresis were performed after each run. The relative expression data to β-actin were calculated according to the 2^-ΔΔCt^ method and presented as fold change to the control. All reactions were assessed in triplicate.

### Homology modeling and molecular docking

The interaction between scFv antibodies and nTcdA or nTcdB was predicted by *in silico* modeling. For this purpose, I-TASSER server service was used to predict the tertiary protein structures of scFvs (https://zhanglab.ccmb.med.umich.edu/I-TASSER/). To get close to native structure of scFvs, 3D structural models predicted by I-TASSER were refined using the ModRefiner service (http://zhanglab.ccmb.med.umich.edu/ModRefiner/). The 3D structural models of TcdA (PDB ID: 7pog) and TcdB (PDB ID: 7n9y) were retrieved from RCSB database (https://www.rcsb.org). The Haddock server service (https://haddock.science.uu.nl/services/HADDOCK2.2/) was used to investigate the mode of interactions between antigen-antibody-refined models. The possible interactions of structural models were decorated and visualized using Pymol software version 1.5.0.1 (http://pymol.findmysoft.com) and LigPlot plus software version 4.5.3. The final model was selected based on the largest cluster size and minimal local energy.

### Statistical analysis

GraphPad Prism 8.0 (GraphPad Software, CA, USA) was used for statistical analysis. Results were calculated and analyzed for statistical significance using the unpaired Student’s *t* test and one-way analysis of variance (ANOVA). The data were presented as mean ± standard deviation (SD) for three independent experiments. Differences were considered statistically significant when *P* < 0.05; *P < 0.05, **P < 0.01, ***P < 0.001, and ****P < 0.0001.

## Data Availability

All data generated or analyzed during this study are included in this published article and the supplemental information at Figshare.
